# Combined impact of elevated arterial stiffness and left ventricular filling pressure on outcomes after off-pump coronary artery bypass grafting

**DOI:** 10.1186/s13019-022-01994-5

**Published:** 2022-10-02

**Authors:** Jae-Sung Choi, Se Jin Oh, Sohee Oh

**Affiliations:** 1 Department of Thoracic and Cardiovascular Surgery, SMG-SNU Boramae Medical Center, Seoul National University College of Medicine, Seoul, Korea; 2 Department of Biostatistics, Boramae Medical Center, Seoul National University College of Medicine, Seoul, Korea

**Keywords:** Pulse wave velocity, Arterial stiffness, Ventricular filling pressure, Diastolic dysfunction, Survival

## Abstract

**Background:**

Brachial-ankle pulse wave velocity (baPWV) and early diastolic transmitral flow velocity to mitral annular tissue velocity (E/e′)—which are markers of arterial stiffness and left ventricular (LV) filling pressure, respectively—have been associated with morbidity and mortality. We investigated their combined impact on postoperative complications and long-term survival of patients undergoing off-pump coronary artery bypass grafting (OPCAB).

**Methods:**

A cohort of 164 patients were divided into four groups: baPWV ≤ 19 m/s and E/e′ ≤ 15 (reference), baPWV > 19 m/s and E/e′ ≤ 15 (high-PWV-only), baPWV ≤ 19 m/s and E/e′ > 15 (high-E/e′-only), and baPWV > 19 m/s and E/e′ > 15 (high-PWV-and-E/e′). After inverse probability treatment weighting adjustment, each group was compared with the reference group to analyze the odds ratios of postoperative complications and the Kaplan–Meier survival curves, and to identify the group representing an independent prognostic predictor.

**Results:**

The median age and follow-up duration were 69 years and 57.2 months, respectively. Both postoperative acute kidney injury (POAKI) and atrial fibrillation (POAF) were higher in the high-PWV-and-E/e′ group (adjusted odds ratio (OR) = 89.5; 95% confidence interval (CI), 8.5–942.3; *p* < 0.001 and OR = 12.5; CI, 2.5–63.8; *p* = 0.002, respectively). Compared to the reference group, only the high-PWV-and-E/e′ group showed significantly lower survival rate (91.0%; CI, 82.8–100% vs. 44.8%; CI, 21.2–94.6%) and a higher hazard for all-cause mortality after adjustment for covariates (hazard ratio = 6.1; *p* = 0.002).

**Conclusion:**

Concurrent elevation in PWV and E/e′ may independently affect not only the rates of POAKI and POAF but also long-term survival after OPCAB.

**Supplementary Information:**

The online version contains supplementary material available at 10.1186/s13019-022-01994-5.

## Background

Pulse wave velocity (PWV) is now widely accepted as an index of arterial stiffness [1]. As increased arterial stiffness may promote left ventricular (LV) hypertrophy and reduce coronary perfusion, elevated PWV may be associated with LV diastolic dysfunction and heart failure with preserved ejection fraction (HFpEF) [2, 3]. The ratio of early transmitral blood flow velocity to early diastolic mitral annular velocity (E/e′) is a marker of filling pressure which has been used to estimate the diastolic function [4]. Like elevated PWV, elevated E/e′ is associated with a higher risk of cardiovascular events and deaths in various patient populations [5, 6]. However, the association of PWV or E/e′ with cardiac surgical outcomes has rarely been reported.

In a previous study, we reported earlier that elevated brachial-ankle PWV (baPWV) was an independent predictor of postoperative acute kidney injury (POAKI) that was also associated with the composite rate of stroke and/or delirium as well as the duration of ventilatory support [7]. In another work, our team demonstrated that high PWV was significantly correlated with elevated E/e′ [8].

Considering the reported negative impact of diastolic dysfunction on post-cardiovascular surgery outcomes [9], we hypothesized that the combined impact of elevated PWV and E/e′ is greater than the impact of the isolated elevation of either PWV or E/e′. We also speculated that this concurrent elevation of PWV and E/e might be a more reliable predictor of long-term survival.

The aim of this study was to investigate whether the combination of significant elevations in both PWV and E/e′ affects postoperative complications and long-term overall survival after off-pump coronary artery bypass grafting (OPCAB).

## Materials and methods

### Patient selection

**Fig. 1:**
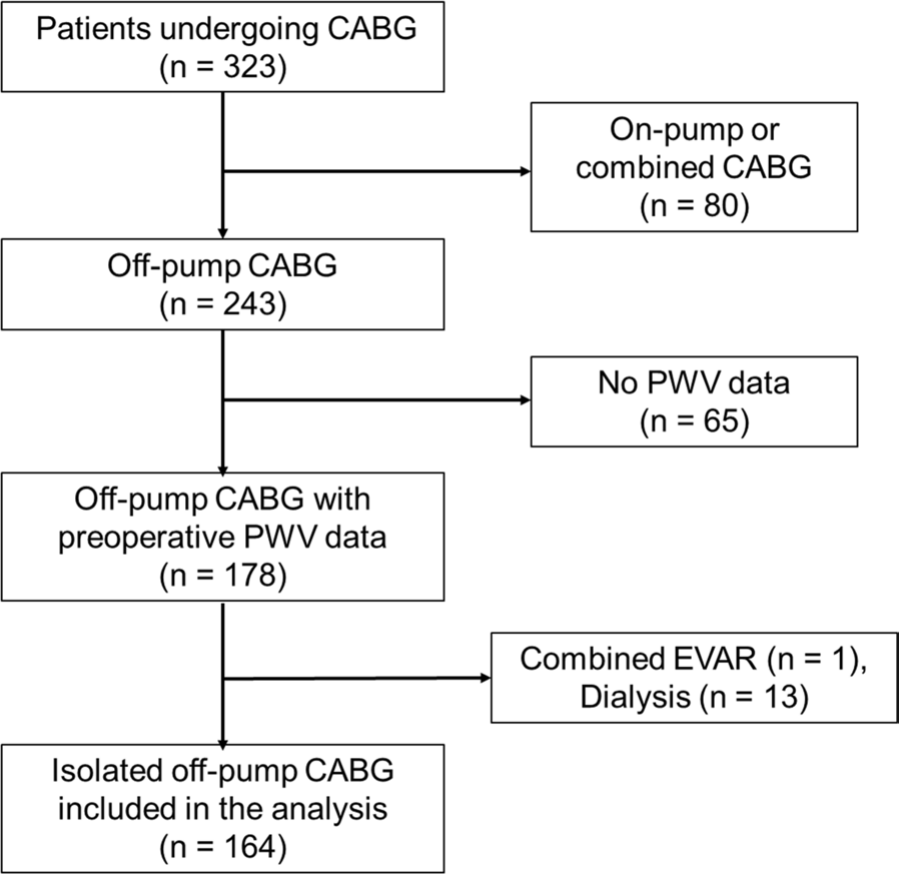
Flow chart for patient selection. CABG, coronary artery bypass grafting; EVAR, endovascular abdominal aortic repair; PWV, pulse wave velocity

This study cohort is similar to our previous one with similar inclusion and exclusion criteria (Fig. 1) [7]. The present work additionally analyzed images of the immediate postoperative coronary angiography (CAG) and the preoperative tissue Doppler echocardiography, and the follow-up of the cohort was lengthened to a median of 57.2 months postoperatively to investigate longer-term survival. In total, 323 patients underwent coronary artery bypass grafting (CABG) between April 2013 and July 2019. From these patients, 159 patients were excluded, and 164 patients undergoing isolated OPCAB were ultimately analyzed. The exclusion criteria were: (1) On-pump CABG, to avoid bias due to cardiopulmonary bypass, (2) absence of PWV measurements, (3) insertion of aorto-iliac or renal stent, (4) patients with oliguria or already undergoing dialysis, (5) combination with any other cardiac procedures, (6) uncontrolled and severe hypertension (blood pressure > 160/100 mmHg), to avoid potentially incorrect assessments of PWV that can change with wild fluctuations of BP.

The study protocol was approved by the Seoul Metropolitan Government—Seoul National University Hospital’s institutional review board (IRB No. 10-2021-140), and the requirement for informed consent was waived because this retrospective review of medical records did not adversely affect the rights or welfare of the subjects.

### Preoperative measurement of baPWV and E/e′

The vast majority of the patients—who were referred for CABG from the cardiology department—underwent PWV measurements prospectively before surgery because several prospective studies related to PWV involved patients with coronary artery disease. The measurement was simple and noninvasive. By wrapping cuffs around both brachialis and ankles, the pulse volume waveform, blood pressure, phonogram, and heart rate were recorded simultaneously using a volume-plethysmographic apparatus (VP-1000, Colin Co. Ltd.; Komaki, Japan). A higher PWV value suggests a stiffer arterial system. The mean values between the left and right baPWVs were used for analysis. We have already previously detailed the calculation of baPWV [7]. Accordingly, high baPWV was defined as baPWV > 19 m/s, which was an independent predictor of POAKI following OPCAB.

Transthoracic echocardiography (Sequoia, Siemens Medical Solutions or Vivid 7, GE Medical Systems) with tissue Doppler analysis was routinely performed before surgery. The peak early transmitral filling velocity during early diastole (E) was imaged and early diastolic velocity at the septal mitral annulus (e′) was determined in the apical four-chamber view, under pulsed-wave Doppler examination of the mitral inflow as well as tissue Doppler imaging of the mitral annulus. We intended to exclude cases in which e′ and E/e′ may not provide a reliable estimate of LV filling pressure due to valve pathogenesis, including significant mitral annular calcification and moderate-to-severe mitral regurgitation, but no such case had to be excluded. Based on the 2016 American Society of Echocardiography (ASE) recommendations for the evaluation of LV diastolic function using echocardiography [10] and studies investigating E/e′ [11], high E/e′ was defined as an E/e′ ratio greater than 15 at the septal side.

### Surgical techniques

Our OPCAB strategy has already been described in our previous study investigating the association between baPWV and POAKI [7]. In brief, we first fabricated the composite graft by attaching the saphenous vein (SV) harvested from the lower leg to the in situ left internal thoracic artery (LITA) in a Y-shaped configuration. Next, the LITA was anastomosed to the left anterior descending coronary artery, followed by SV sequential anastomoses to the other target coronary arteries. For cases in which a single inflow source was not appropriate due to flow competition, the proximal end of the SV graft was attached to the proximal ascending aorta via partial clamping or by using a Heartstring III proximal seal system (Maquet holding B.V. & Co., Rastatt, Germany). A total arterial bypass was occasionally performed using either the right internal thoracic artery or the radial artery in patients younger than 60 years of age. All patients routinely underwent immediate postoperative CAG to confirm the quality of bypass anastomoses. When the problems that were found correctible, such as in cases of graft kinking, thrombosis, or occlusion, they were addressed by adjuvant percutaneous coronary intervention or reopening sternum followed by the release of kinking or additional bypass grafting.

### Definitions of postoperative and long-term outcomes

Operative mortality was defined as the number of deaths within 30 days of surgery. Daily 12-lead electrocardiogram (ECG) and continuous monitoring of the ECG were routinely performed. Levels of cardiac enzymes such as creatine kinase isoenzyme (CK-MB) and troponin I were routinely assessed at 1, 12, 24, and 48 h after protamine reversal. Perioperative myocardial infarction (PMI) was defined by an elevation in biomarkers including either CK-MB concentration > 40 ng/mL or peak troponin I levels > 15 ng/mL at 12 h postoperatively along with the presence of new pathological Q waves or left bundle branch block at ECG. Renal function was assessed by measuring the levels of serum creatinine and calculating the estimated glomerular filtration rate (eGFR) using the Modification of Diet in Renal Disease (MDRD) equation. Postoperative GFR was defined as the lowest eGFR within 7 postoperative days (PODs). According to the 2012 Kidney Disease: Improving Global Outcomes (KDIGO) Foundation consensus statement [12], POAKI was defined as stage 1 or higher based on any of the following criteria: urine output < 0.5 mL/kg/h for 6 h or longer; elevation of serum creatinine within 2 PODs > 0.3 mg/dL; and a serum creatinine increase > 1.5-fold relative to the baseline value within 7 PODs. Postoperative stroke/delirium (POSD) was a composite variable consisting of isolated stroke and isolated delirium, and a combination thereof. Stroke was defined as cerebral infarction of ischemic or hemorrhagic etiology, or transient ischemic attack based on brain imaging studies and consultation with neurologists. Delirium was defined based on the criteria of the fifth edition of the Diagnostic and Statistical Manual of the American Psychiatric Association (DSM-5) and the diagnosis was established in consultation with neuropsychiatrists. Postoperative pneumonia was defined as a lower respiratory tract infection with accompanying consolidation detected on a chest X-ray.

Late mortality was defined as all-cause mortalities after 30 days of operation. Patients were identified as still living on October 31, 2021. The median follow-up duration was 57.2 months (range, 40.0–84.0 months) and none of the patients were lost to follow-up.

### Statistical analysis

For descriptive statistics, the categorical variables are expressed as counts and percentages. Values for continuous variables are expressed as mean ± standard deviation (SD) or, when the data are not equally distributed, as median (interquartile range, IQR). The baseline characteristics and postoperative complications were descriptively analyzed and compared across the four independent groups. One-way analysis of variance (ANOVA) or the Kruskal–Wallis test was used to test differences across the groups according to the normality assumption, which was assessed with the Kolmogorov–Smirnov test. Post-hoc analysis was also performed using Bonferroni’s t-test or Dunn’s test for normally distributed or non-normally distributed continuous variables, respectively. For categorical variables, the Pearson *χ*2 test or the Fisher’s exact test was used. Further, to address the selection bias between groups, the inverse probability of treatment weighting (IPTW) was performed, which was estimated as the inverse of propensity scores. The propensity score was derived from a generalized logit model with demographic variables such as sex, age, body mass index (BMI), and smoking history.

Multivariable logistic regression models, which were adjusted for baseline characteristics including preexisting comorbidities, were used to compare the odds ratios of each group with those of the reference group to analyze the several important postoperative complications. Survival rate was estimated using Kaplan–Meier methods and between-group comparisons were performed using the log-rank test. Univariable and multivariable Cox proportional-hazards models were used to evaluate the impact of the risk factors on overall survival. The covariates, which were included in the multivariable logistic or Cox regression models, were selected for having exhibited statistical significance in the univariable analyses. A *p*-value < 0.05 was considered to be statistically significant. For multiple inter-group comparisons, Bonferroni correction was applied. Analyses were performed using IBM SPSS Statistics for Windows, version 26.0 (IBM Corp., Armonk, NY, USA) and R version 4.1.1 (R Foundation for Statistical Computing, Vienna, Austria).

## Results

### Baseline characteristics

The median age of this study population was 69 years and 79.9% of the patients had triple-vessel disease, with a median EF of 61.0% and a median EuroSCORE II value of 1.6. To assess the effect of each group on the outcomes, the patients were divided into four groups: (1) elevation in baPWV alone (baPWV > 19 m/s and E/e′ ≤ 15, high-PWV-only group), (2) elevation in E/e′ alone (baPWV ≤ 19 m/s and E/e′ > 15, high-E/e′-only group), (3) elevation in both baPWV and E/e′ (baPWV > 19 m/s and E/e′ > 15, high-PWV-and-E/e′ group), and elevation in neither baPWV nor E/e′ (baPWV ≤ 19 m/s and E/e′ ≤ 15, reference group). In terms of the potential influence of mitral regurgitation (MR) on E/e′, such an influence was expected to be minimal, as all the patients with MR > moderate grade underwent combined mitral procedure and were therefore excluded from this study in advance. It can be seen in Table 1 that the differences between groups in age, sex, and smoking history disappeared after IPTW adjustment. The parameters of hypertension, diabetes, EF, eGFR,, EuroSCORE II, and CHA2DS2-VASc SCORE differed across the four groups: hypertension rate, diabetes rate, and CHA2DS2-VASc SCORE were all highest in the high-PWV-and-E/e′ group. LVEF was significantly low in the high-E/e′-only group because 17 patients (58.6%) with EF < 50% were included in this group. EuroSCORE II value was the highest in the high-E/e′-only group. Estimated GFR was the lowest in the high-PWV-and-E/e′ group. However, the groups did not show differences in the frequencies of cerebrovascular accidents, peripheral arteriopathy, acute myocardial infarction, extent of coronary vessel involvement including left main disease, and previous coronary intervention.

**Table 1:** Patient characteristics

	Before IPTW adjustment						After IPTW adjustment				
	Total	Reference^*a*^	High-PWV -only	High-E/e′ -only	High-PWV -and-E/e′	*P*	Reference	High-PWV -only	High-E/e′ -only	High-PWV -and-E/e′	*P*
N	164	89	28	29	18		89	28	29	18	
Age, years	69.0 (60.0–73.8)	63.0 (56.0–70.0)	71.0 (66.0–77.0)	70.0 (61.0–75.8)	72.5 (69.3–79.0)	< 0.001	69.0 (60.0–73.0)	69.0 (64.0–74.0)	69.0 (58.0–75.0)	67.0 (59.0–72.0)	0.473
*Age > 75 years*, n (%) or %	27 (16.5)	4 (4.5)	7 (25.0)	8 (27.6)	8 (44.4)	< 0.001	10.1	14.5	22.0	19.7	0.352
Female, n (%) or %	116 (70.7)	72 (80.9)	18 (64.3)	19 (65.5)	7 (38.9)	0.003	71.3	64.4	68.3	66.6	0.915
Body mass index, kg/m^2^	24.2 ± 3.2	24.7 ± 3.0	23.8 ± 3.1	22.9 ± 3.4	24.0 ± 3.7	0.056	24.1 ± 3.2	23.9 ± 3.3	24.4 ± 3.6	24.0 ± 3.1	0.954
Smoking, n (%) or %	84 (51.2)	54 (60.7)	11 (39.3)	14 (48.3)	5 (27.8)	0.032	54.4	47.7	50.0	58.8	0.861
Hypertension, n (%) or %	117 (71.3)	58 (65.2)	21 (75.0)	21 (72.4)	17 (94.4)	0.087	68.9	76.4	61.9	98.2	0.034
Diabetes, n (%) or %	87 (53.0)	35 (39.3)	23 (82.1)	14 (48.3)	15 (83.3)	< 0.001	37.9	89.1	41.9	91.6	< 0.001
*Under insulin therapy, n (%) or %*	*24 (14.6)*	4 (4.5)	11 (39.3)	5 (17.2)	4 (22.2)	< 0.001	3.4	40.3	12.2	13.3	< 0.001
Dyslipidemia, n (%) or %	54 (32.9)	28 (31.5)	13 (46.4)	7 (24.1)	6 (33.3)	0.332	28.5	48.5	22.3	27.7	0.172
COPD, n (%) or %	10 (6.1)	4 (4.5)	1 (3.6)	3 (10.3)	2 (11.1)	0.450	4.8	1.9	9.1	9.5	0.577
CVA, n (%) or %	26 (15.9)	9 (10.1)	4 (14.3)	8 (27.6)	5 (27.8)	0.059	11.9	14.4	28.4	31.4	0.068
PAD, n (%) or %	45 (27.4)	21 (23.6)	9 (32.1)	8 (27.6)	7 (38.9)	0.502	26.3	27.3	23.2	35.4	0.821
AMI, n (%) or %	24 (14.6)	12 (13.5)	4 (14.3)	5 (17.2)	3 (16.7)	0.933	15.1	14.0	13.0	16.7	0.985
Triple-vessel disease, n (%) or %	131 (79.9)	71 (79.8)	20 (71.4)	26 (89.7)	14 (77.8)	0.377	75.3	82.0	82.9	52.8	0.084
Left main disease, n (%) or %	56 (34.1)	29 (32.6)	11 (39.3)	11 (37.9)	5 (27.8)	0.817	30.8	36.9	47.5	29.4	0.377
Previous PCI, n (%) or %	22 (13.4)	13 (14.6)	5 (17.9)	2 (6.9)	2 (11.1)	0.638	14.6	14.4	4.9	13.9	0.562
Atrial fibrillation, n (%) or %	2 (1.2)	0 (0.0)	0 (0.0)	1 (3.4)	1 (5.6)	0.147	0.0	0.0	2.0	2.5	0.457
Left ventricular EF, %	61.0 (51.0–66.6)	61.7 (56.7–66.5)	62.1 (57.0–67.5)	47.2 (30.3–61.8)	66.2 (60.0–67.8)	< 0.001	62.2 (55.0–66.8)	62.0 (54.0–66.0)	46.2 (28.0–58.9)	66.4 (60.0–69.0)	< 0.001
MDRD-GFR, mL/min/1.73 m^2^	83.1 (66.5–95.9)	88.2 (76.5–97.9)	80.2 (48.7–102.6)	76.6 (52.1–92.5)	67.6 (47.5–81.9)	0.002	87.1 (75.6–96.4)	78.6 (49.6–100.0)	78.2 (54.8–92.6)	75.6 (51.8–91.3)	0.021
CKD stage ≥ 4, n (%) or %	20 (12.2)	5 (5.6)	4 (14.3)	7 (24.1)	4 (22.2)	0.015	4.8	11.4	16.6	20.8	0.082
Mean baPWV, m/s	16.3 (14.2–19.3)	15.0 (13.3–16.7)	20.6 (19.8–21.6)	15.2 (13.5–16.9)	22.8 (20.2–23.8)	< 0.001	15.2 (13.6–16.8)	20.6 (19.9–21.6)	15.7 (13.5–16.9)	22.1 (19.6–23.9)	< 0.001
*Right baPWV, m/s*	16.50 (14.2–19.5)	15.1 (13.6–17.0)	20.9 (19.7–22.1)	15.4 (13.8–17.1)	22.0 (20.7–24.2)	< 0.001	15.1 (13.6–17.2)	20.9 (19.8–21.8)	15.5 (13.8–17.5)	21.9 (18.9–24.3)	< 0.001
*Left baPWV, m/s*	16.3 (14.2–19.5)	15.2 (13.1–16.7)	20.6 (19.6–22.2)	14.9 (13.4–16.7)	23.6 (21.6–24.5)	< 0.001	15.6 (13.3–16.8)	20.3 (19.7–22.2)	15.1 (13.3–17.0)	22.3 (19.8–24.8)	< 0.001
E, cm/s	59.0 (50.0–71.0)	56.0 (49.0–64.0)	58.0 (47.0–65.0)	79.0 (65.0–98.8)	70.5 (52.8–93.8)	< 0.001	55.0 (49.0–64.0)	57.0 (43.0–65.0)	72.0 (65.0–98.0)	62.0 (49.0–88.0)	< 0.001
A, cm/s	82.0 (67.7–97.0)	72.0 (64.0–86.0)	91.0 (74.0–97.0)	94.5 (61.8–115.0)	108.0 (84.0–122.0)	< 0.001	74.0 (65.0–89.0)	90.0 (73.0–96.0)	87.0 (57.0–104.0)	91.0 (68.0–112.0)	0.960
E/A	0.7 (0.6–0.9)	0.8 (0.6–0.9)	0.6 (0.6–0.7)	0.8 (0.6–1.6)	0.7 (0.7–0.9)	0.004	0.7 (0.6–0.9)	0.6 (0.6–0.7)	0.8 (0.6–1.6)	0.7 (0.7–0.9)	< 0.001
e′, cm/s	5.0 (4.0–6.0)	5.0 (4.0–7.0)	5.0 (4.0–6.0)	4.0 (3.0–5.0)	4.0 (3.0–5.0)	< 0.001	5.0 (4.0–6.0)	5.0 (4.0–6.0)	4.0 (3.0–4.0)	4.0 (3.0–4.0)	< 0.001
E/e′	12.2 (9.6–16.5)	10.8 (8.7–12.5)	10.8 (9.6–14.0)	18.8 (16.9–23.9)	19.9 (17.5–23.5)	< 0.001	10.8 (8.5–12.5)	10.4 (9.0–12.8)	18.7 (17.0–24.0)	18.0 (16.0–20.4)	< 0.001
EuroSCORE II	1.6 (0.9–2.9)	1.0 (0.8–1.7)	1.9 (1.4–3.2)	2.8 (1.5–4.4)	1.9 (1.6–3.2)	< 0.001	1.4 (0.8–2.8)	1.9 (1.1–2.9)	2.5 (1.4–3.5)	1.6 (1.3–2.0)	0.023
*EuroSCORE II > 4, n (%) or %*	24 (14.6)	7 (7.9)	5 (17.9)	9 (31.0)	3 (16.7)	0.018	10.2	13.1	20.8	6.1	0.373
CHA2DS2-VASc SCORE	4 (2–5)	3 (2–4)	5 (4–5)	4 (3–6)	6 (5–7)	< 0.001	3 (2–5)	5 (4–5)	4 (2–5)	6 (5–22)	< 0.001

*A* late diastolic mitral inflow velocity; *AMI* acute myocardial infarction; *baPWV* brachial-ankle pulse wave velocity; *CKD* chronic kidney disease; *COPD* chronic obstructive pulmonary disease; *CVA* cerebrovascular accident; *E* early diastolic mitral inflow velocity; *e′* early diastolic mitral annular tissue velocity; *GFR* glomerular filtration rate; *IPTW* inverse probability of treatment weighting; *PAD* peripheral arteriopathy

Italics indicate nested data

^a^Reference: neither high PWV nor high E/e′

### Postoperative outcome

Table 2 presents the statistics of postoperative outcomes. There was no operative mortality. The PMI rate was 9.8%, which was slightly higher, but it was not different across the groups. It can at least said that this higher rate was not related to more frequent graft occlusion, because total graft patency based on the immediate postoperative coronary angiographic findings was nearly 97%, which was not bad; moreover, the numbers of the patients with PMI and the patients with the graft occlusion were not matched, as there were 15 patients with at least one graft occlusion and only four patients with PMI. The complication variables demonstrating significant differences across the groups were POAKI (*p* < 0.001), postoperative atrial fibrillation (POAF) (*p* < 0.001), and intra-aortic balloon pump support (*p* = 0.001). Table 3 presents the impact of combined elevation of PWV and E/e′ on major postoperative complications after adjustment for various pre-existing comorbidities. Compared to the reference group, only the high-PWV-and-E/e′ group showed a statistically higher adjusted odds ratio (OR) (OR = 89.5; 95% confidence interval (CI), 8.5–942.3; *p* < 0.001). The high-PWV-only group showed a trend of POAKI although the *p*-value was higher than 0.05. The adjusted OR of POAF was also significantly higher only in the high-PWV-and-E/e′ group (OR = 12.5; CI, 2.5–63.8; *p* = 0.002). There were no differences in POSD risk among these inter-group comparisons after adjusting for multiple covariates. For reference, the results of the analysis before IPTW adjustment were summarized in Additional file 1: Table S1 and S2).

**Table 2:** Postoperative complications

	After IPTW adjustment				
	Reference^*a*^	High-PWV -only	High-E/e′ -only	High-PWV -and-E/e′	*P*
N	89	28	29	18	
AKI, n (%) or %	5.4	19.8	7.1	52.9	< 0.001^*c,e*^
Stroke/delirium, n (%) or %	12.9	15.9	5.8	24.0	0.319
Atrial fibrillation, n (%) or %	23.1	7.8	27.9	68.6	< 0.001^*c,d,e*^
Graft patency, PA/DA (%) or %	97.7	94.7	96.6	98.1	0.745
Perioperative MI, n (%) or %	10.4	16.8	3.3	0.0	0.158
IABP support, n (%) or %	0.8	0.0	15.7	0.0	0.001^*b*^
ECMO support, n	0	0	0	0	–
Pneumonia, n (%) or %	4.4	4.3	13.3	9.5	0.343
Peak troponin-I, ng/mL	2.5 (1.0–5.2)	2.2 (1.3–5.4)	1.3 (0.6–2.6)	3.4 (1.1–5.7)	0.373
Ventilator support, hrs	17.7 (14.0–21.0)	18.5 (15.0–23.7)	18.9 (15.0–23.7)	22.0 (19.0–25.6)	0.533
ICU stay, hrs	46.0 (28.0–69.5)	46.0 (26.0–91.0)	45.0 (26.0–91.0)	46.0 (37.0–67.0)	0.807
Hospital stay, days	9.0 (8.0–12.0)	9.0 (8.0–12.0)	9.0 (8.0–12.0)	9.0 (9.0–26.0)	0.079
30-day mortality, n	0	0	0	0	–

*AKI* acute kidney injury; *DA* total number of distal anastomoses; *E* early diastolic mitral inflow velocity; *e′* early diastolic mitral annular tissue velocity; *ECMO* extracorporeal membrane oxygenator; *IABP* intraaortic balloon pump; *ICU* intensive care unit; *IPTW* inverse probability of treatment weighting; *MI* myocardial infarction; *PA* total number of patent anastomoses; *PWV* pulse wave velocity

^*a*^Reference: neither high PWV nor high E/e′

Post-hoc tests were performed between the following groups: ^*b*^High-E/e'-only vs. Reference; ^*c*^High-PWV-and-E/e' vs. Reference; ^*d*^High-PWV-and-E/e' vs. High-PWV-only; ^*e*^High-PWV-and-E/e' vs. High-E/e'-only; ^*f*^High-PWV-only vs. Reference; ^*g*^High-E/e'-only vs. High-PWV-only

**Table 3:** Comparison of major postoperative complications across the groups

	n (%)	After IPTW adjustment	
		OR (95% CI)	*P*
POAKI	26 (15.9)		0.002
Reference^*a*^	5 (5.6)	1	
High-PWV-only	7 (25.0)	6.193 (0.987, 38.860)	0.052
High-E/e′-only	3 (10.3)	0.963 (0.112, 8.285)	0.973
High-PWV-and-E/e′	11 (61.1)	89.458 (8.493, 942.275)	< 0.001
		Hosmer–Lemeshow test	0.7663
POAF	45 (27.4)		0.004
Reference	21 (23.6)	1	
High-PWV-only	2 (7.1)	0.321 (0.053, 1.937)	0.216
High-E/e′-only	11 (37.9)	2.119 (0.645, 6.958)	0.216
High-PWV-and-E/e′	11 (61.1)	12.512 (2.453, 63.830)	0.002
		Hosmer–Lemeshow test	0.091
POSD	19 (11.6)		0.736
Reference	8 (9.0)	1	
High-PWV-only	4 (14.3)	1.315 (0.278, 5.491)	0.718
High-E/e′-only	2 (6.9)	0.566 (0.083, 2.623)	0.483
High-PWV-and-E/e′	5 (27.8)	1.744 (0.296, 10.510)	0.523
		Hosmer–Lemeshow test	0.480

*CI* confidence interval; *E* early diastolic mitral inflow velocity; *e′* early diastolic mitral annular tissue velocity; *IPTW* inverse probability of treatment weighting; *OR* odds ratio; *POAF* postoperative atrial fibrillation; *POAKI* postoperative acute kidney injury; *POSD* postoperative stroke and/or delirium; *PWV* pulse wave velocity

^a^Reference: neither high PWV nor high E/e′

### Late mortality and long-term survival

**Fig. 2:**
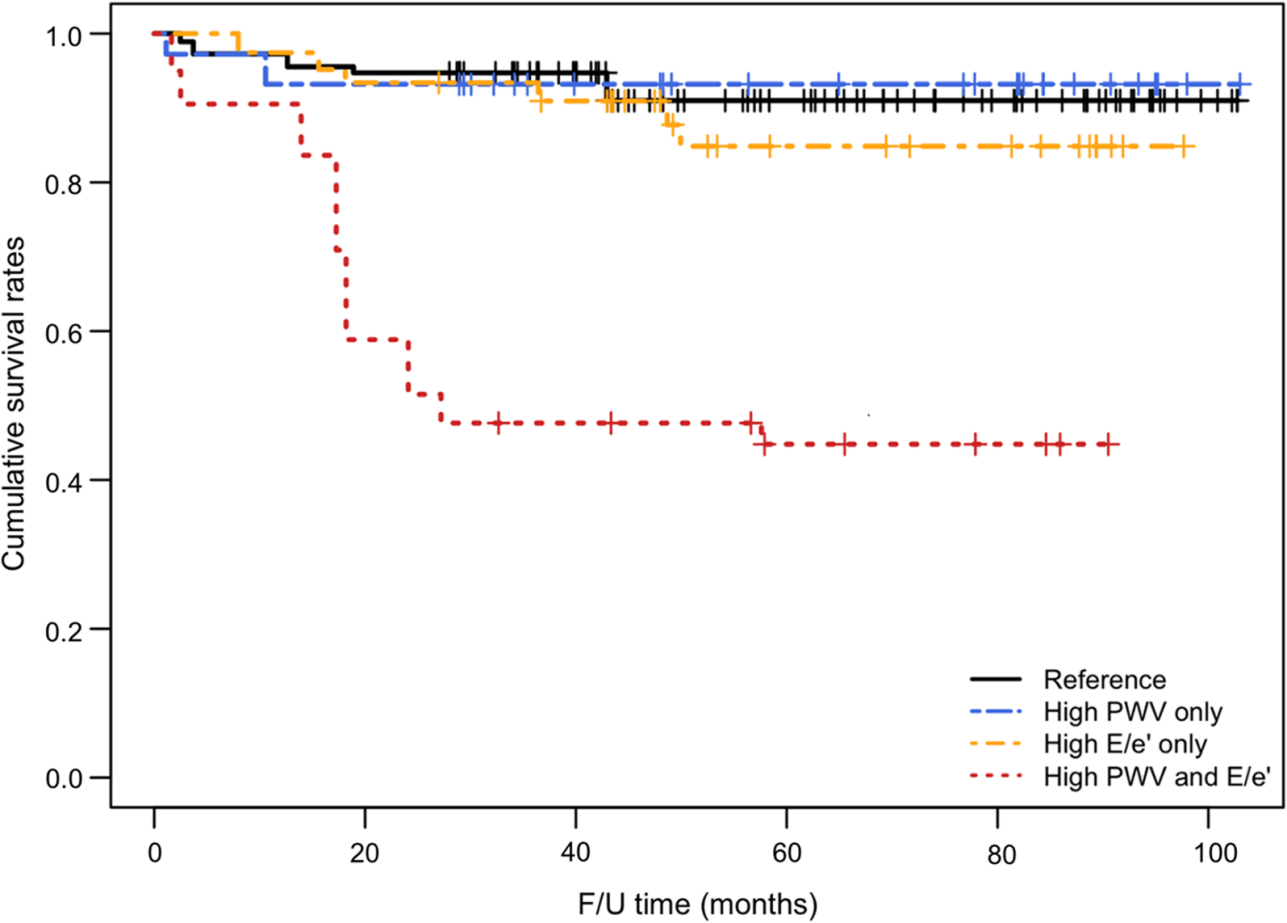
Comparison of Kaplan–Meier overall survival curves

Late all-cause mortality of the patient cohort was 12.8% (21 of 164 patients). The late mortalities were 5.6%, 7.1%, 20.7%, and 44.4% for the reference, high-PWV-only, high-E/e′-only, and high-PWV-and-E/e′ group, respectively. After IPTW adjustment, the unadjusted estimated overall survivals of respective groups are presented as Kaplan–Meier curves in Fig. 2. The overall survival rates differed significantly across the groups (*p* < 0.001), as they were 91.0% (CI, 82.8–100%), 93.2% (CI, 84.3–100%), 84.9% (CI, 73.2–98.4%), and 44.8% (CI, 21.2–94.6%) in the reference, high-PWV-only, high-E/e′-only, and high-PWV-and-E/e′ group, respectively. The overall survivals of the high-PWV-only and high-E/e′-only groups were not different from that of the reference group in the inter-group comparisons (Table 4). Only the survival rate of the high-PWV-and-E/e′ group was significantly lower, and this significance was maintained even after covariate adjustment (HR = 6.1 (CI, 2.0–18.5); Bonferroni adjusted *p* = 0.009). Although the preoperative LVEF and EuroSCORE II values were worst in the high-E/e′-only group, the HRs of these values turned out to be insignificant for all-cause mortality. (, respectively).) (Table 4). We also found that cerebrovascular accident (CVA) and chronic obstructive pulmonary disease (COPD) were independent risk factors for all-cause mortality (HR = 2.8; CI, 1.1–7.2; *p* = 0.034 and HR = 3.8; CI, 1.1–13.8; *p* = 0.040) (Table 5). For reference, the results of the analysis before IPTW adjustment were summarized in Additional file 1: Tables S3 and S4).

**Table 4:** Inter-group comparison with Cox regression analysis for all-cause mortality

Group comparison	After IPTW adjustment			
	Univariable analysis		Multivariable analysis	
	HR (95% CI)	*P*^*b*^	HR (95% CI)	*P*^*b*^
Group		< 0.001		0.004
High-PWV-only vs. Reference^*a*^	0.866 (0.163, 4.614)	1.000	0.882 (0.163, 4.788)	1.000
High-E/e′-only vs. Reference	1.596 (0.473, 5.387)	1.000	0.984 (0.275, 3.528)	1.000
High-PWV-and-E/e′ vs. Reference	8.466 (3.262, 21.971)	0.005	6.090 (2.001, 18.532)	0.009
High-E/e′-only vs. High-PWV-only	1.843 (0.306, 11.099)	1.000	1.116 (0.174, 7.168)	1.000
High -PWV-and-E/e′ vs. High-PWV-only	9.777 (1.919, 49.806)	0.037	6.903 (1.247, 38.215)	0.161
High-PWV-and-E/e′ vs. High-E/e′-only	5.304 (1.672, 16.828)	0.028	6.186 (1.693, 22.598)	0.035

*CI* confidence interval; *E* early diastolic mitral inflow velocity; *e′* early diastolic mitral annular tissue velocity; *HR* hazard ratio; *IPTW* inverse probability of treatment weighting; *PWV* pulse wave velocity

^*a*^Reference: neither high PWV nor high E/e′; ^*b*^Bonferroni adjusted p-value

**Table 5:** Analysis of risk factors for all-cause mortality with Cox proportional hazards model

Risk factors	After IPTW adjustment			
	Univariable analysis		Multivariable analysis	
	HR (95% CI)	*P*	HR (95% CI)	*P*^*b*^
Group		< 0.001		0.004
*Reference*^*a*^	1		1	
*vs. High-PWV-only*	0.866 (0.163, 4.614)	0.866	0.882 (0.163, 4.788)	0.885
*vs. High-E/e′-only*	1.596 (0.473, 5.387)	0.451	0.984 (0.275, 3.528)	0.981
*vs. High-PWV-and-E/e′*	8.466 (3.262, 21.971)	< 0.001	6.090 (2.001, 18.532)	0.002
Sex, Female	1.081 (0.447, 2.616)	0.863		
Age	1.044(0.994 1.097)	0.085		
Age 75	2.421 (0.972, 6.027)	0.058		
Body mass index	0.886 (0.783, 1.002)	0.054		
Smoking	1.303 (0.570, 2.980)	0.531		
Obesity	0.389 (0.147, 1.028)	0.057		
NYHA functional class	0.829 (0.449, 1.531)	0.549		
Hypertension	3.409 (0.933, 12.454)	0.064		
Diabetes	1.520 (0.659, 3.507)	0.326		
Insulin	1.792 (0.615, 5.218)	0.285		
Dyslipidemia	1.854 (0.813, 4.228)	0.142		
Cerebrovascular accident	3.172 (1.368, 7.356)	0.007	2.796 (1.081, 7.233)	0.034
Peripheral arteriopathy	2.814 (1.248, 6.347)	0.013	1.867 (0.731, 4.772)	0.192
CKD grade ≥ 4	4.073 (1.640, 10.115)	0.003	2.611 (0.872, 7.823)	0.086
COPD	3.275 (1.003, 10.697)	0.049	3.830 (1.061, 13.833)	0.040
Acute myocardial infarction	1.526 (0.561, 4.149)	0.408		
Atrial fibrillation	2.930 (0.167, 51.358)	0.462		
PCI	1.028 (0.289, 3.657)	0.966		
EuroSCORE II	1.088 (0.965, 1.228)	0.170		
*EuroSCORE II ≥ 4*	1.432 (0.487, 1.211)	0.514		
*EuroSCORE II > 7*	2.154 (0.362, 12.809)	0.399		
CHA2DS2-VASc SCORE	1.081 (1.016, 1.151)	0.013	1.023 (0.941, 1.111)	0.597
Ejection fraction	1.011 (0.979, 1.044)	0.514		
Pulmonary hypertension	1.089 (0.989, 1.199)	0.083		
Preoperative IABP	0.405 (0.023, 7.090)	0.536		
Three vessel disease	0.537 (0.232, 1.245)	0.147		
Left main disease	0.841 (0.351, 2.013)	0.697		
Urgency	0.989 (0.096, 10.232)	0.992		

*CI* confidence interval; *CKD* chronic kidney disease; *E* early diastolic mitral inflow velocity; *e′* early diastolic mitral annular tissue velocity; *HR* hazard ratio; *IABP* intraaortic balloon pump; *IPTW* inverse probability of treatment weighting; *OR* odds ratio; *PCI* percutaneous coronary intervention; *PWV* pulse wave velocity

Italics indicate nested data

^*a*^Reference: neither high PWV nor high E/e′; ^*b*^The covariates included in the multivariable models were selected based on statistical significance (*p* < 0.05) in univariable analysis

## Discussion

We demonstrated that the concurrent elevation of PWV and E/e′ influenced POAKI, POAF and, critically, long-term survival in patients undergoing OPCAB, even after adjusting for potential risk factors. However, the isolated elevation of each indicator did not significantly affect the postoperative outcomes. This implies that the various pathways to the postoperative outcomes from the two indicators—PWV and E/e′—were integrated to take effect. It can therefore be inferred that the numbers of patients with concurrent elevation in the two indicators, with high possibility, were unwittingly included in most of the previous data separately demonstrating the predictive value of either indicator. One example of this can be seen in our previous study with the same cohort, which elucidated that baPWV was an independent predictor of POAKI. Although the authors were not aware of the additional impact of E/e′ at that point, it is true that the 18 cases with the concurrent elevation were embedded in the high baPWV group [7].

Due to the absence of early mortality in this study, the ways in which arterial stiffness and diastolic filling pressure affect early mortality could not be analyzed. However, POAKI and POAF were significantly affected by the concurrent elevation of PWV and E/e′. Whether these complications are related to early cardiovascular mortality in this kind of population requiresfurther investigation [13, 14]. Partially consistent with earlier studies [15] and our previous report [7], isolated high PWV here showed a trend of being an independent risk factor associated with POAKI (OR = 6.2; *p* = 0.052). However, the combination of elevated E/e′ and high PWV led to a substantial increase in the odds ratio of POAKI (OR, 89.5; *p* < 0.001), even though the isolated high of E/e′ was not associated with POAKI. By contrast, a recent retrospective observational study revealed that an E/e′ > 15 was an independent predictor of POAKI occurring after OPCAB [11]; it should be noted that patients with concurrently high PWV and E/e′ could have been unwittingly included in that E/e′ > 15 group though. However, there have been some explanations of the association between high E/e′ and POAKI: First, high E/e′ indicates elevated LV filling pressure and may contribute to increased intra-abdominal venous pressure and subsequent reduction in renal blood flow and GFR [16]. Second, increased LV filling pressure aggravates hemodynamic instability, which is frequently triggered by lifting the heart during OPCAB, which in turn causes renal ischemia. Third, fluid overload during and after operation can induce capillary dysfunction at the glomerulus and may also trigger POAKI. In fact, high PWV is linked to elevated E/e′, which affects postoperative renal function. Increased arterial stiffness triggers premature return of reflected pulse waves, which facilitates diastolic coronary artery filling during late systole [17]. This premature return decreases diastolic pressure and compromises coronary blood flow, which can aggravate the ventricular relaxation disturbance and elevate the LV filling pressure [18].

The results of this study also emphasized the combined impact of elevated arterial stiffness and LV filling pressure on POAF. The isolated high-PWV-only and high-E/e′-only group failed to show statistical significance in predicting POAF, whereas the high PWV-and-E/e′ group did demonstrate significance. In fact, some reports have shown an association either between AF and PWV or between AF and E/e′. PWV has been reported to be significantly correlated with left atrial dimension independent of typical determinants such as age, gender, body mass index, ventricular remodeling and filling pressure, and it is therefore associated with the risk of AF [19]. The broad association between diastolic dysfunction and AF has also been investigated [20]. Current reviews suggest that left atrial and pulmonary vein pressure overload caused by non-compliant LV lead to atrial myocardial remodeling both electrically and structurally, which increases the risk of AF [21]. However, these reports did not investigate either the combined effect or the isolated effect of PWV and E/e′ on AF.

This study chose POSD as a neurologic outcome variable because delirium is one of the afflicting complications that we could come across often after cardiac surgery, and, it is a form of neurocognitive decline that should be treated as brain damage after cardiac surgery together with stroke. Delirium is likely the most common symptom of Type-II brain injuries [22, 23]. High PWV can elicit elevated pulse pressures, which are correlated with stroke [24]. The carotid-femoral PWV exhibited poor neurocognitive dysfunction after aortic valve replacement [25]. However, in this study, POSD was associated with neither the high-PWV-only group nor the high-PWV-and-E/e′ group. As shown in Fig. 2, the long-term survival rate of the high-PWV-and-E/e′ group was significantly higher than that of any other group; with covariates adjustment, the disadvantage in survival was still maintained compared to the reference and the High-E/e′-only group. However, there were no survival differences among the other three groups. In other words, the elevation of a single indicator—among either PWV or E/e′—was not sufficient to affect the survival rate in this study. The two indicators are associated with each other in terms of diastolic dysfunction [8], and each of them has been reported to be associated with all-cause mortalities in various populations. In some large-scale cohort studies with follow-up periods longer than 8 years, PWV has been shown to be associated with all-cause mortality and MI in patients with stable angina, while also having been shown to be associated with cardiovascular mortalities in patients with type 2 diabetes [26, 27]. Regarding the negative impact of E/e′, the increase in LV filling pressure, as assessed by E/e′, was associated with increasing death rate in a cohort of 577 patients undergoing CABG, aortic valve replacement (AVR), or CABG with AVR [6]. The prognostic implications of E/e′ have mainly been investigated in non-surgical patients with various heart diseases. In a study enrolling 230 patients with non-valvular AF, the cumulative survival during follow-up (average 245 days) was significantly lower in subjects with E/e′ > 15 than it was in those with E/e′ ≤ 15, and the high E/e′ was an independent predictor of mortality [28].

Although there have been no robust explanations or hypotheses regarding the synergistic effect of the concurrent elevation of PWV and E/e′ on mortality, it is inferred that different deleterious processes of arterial stiffness and diastolic dysfunction could combine to increase the impact on mortality. For example, in a case of the concurrent elevation of both indicators, there would be an increased possibility of the development of both HFpEF and diastolic dysfunction, which would consequently lead to lower survival, compared to a case of the lone elevation of either indicator. Further, elevated arterial stiffness has other pathophysiology for long-term mortality such as stroke and coronary events [29], which could in turn contribute to the influence of the lone elevation of E/e′.

This study has some limitations that must be addressed. First, the four study groups still do not appear to be balanced, even after IPTW adjustment for demographic data such as age, sex, and smoking history. However, it seems natural that the variables of hypertension and diabetes still differed across the groups because they are all closely related to PWV. Subsequently, these unmatched variables including risk scores were all included in the adjustment for the multivariable regression analyses. Second, the study neither routinely evaluated PWV and E/e′ nor conducted such evaluations in consecutive patients. This might have contributed to selection bias. Third, the study did not consider the potential effect of antihypertensive drugs or hemodynamic alteration on E/e′ and PWV. Moreover, perioperative hemodynamic status and transfusion volume, which could have affected the postoperative renal function, were not controlled. Fourth, unfortunately, baPWV was not routinely assessed postoperatively, and it is not clear whether the two indicators improve after OPCAB and play a role in the improved outcomes; it should be noted that there has been a paucity of data for reference on this subject. Lastly, the sample size was small for a four-group comparison, and the study was retrospective, thus suggesting the need for a larger, prospective study to corroborate or further investigate the findings reported here. Nevertheless, the combined data of PWV and E/e′ in this study represent a rare finding that shows the potential of their promising role in risk prediction, at least in patients undergoing CABG.

## Conclusions

Concurrent elevation in arterial stiffness and LV filling pressure may independently affect not only the rates of major postoperative complications including POAKI and POAF but also long-term survival after OPCAB. The addition of PWV and E/e′ to the baseline model containing conventional risk factors may improve the risk prediction of POAK, POAF, and all-cause mortality. Large-scale studies are needed to further investigate these preliminary findings.

## Supplementary Information

**Additional file 1: Table S1.** Postoperative complications. **Table S2.** Comparison of major postoperative complications across the groups. **Table S3.** Inter-group comparison with Cox regression analysis for all-cause mortality. **Table S4.** Cox regression analysis for all-cause mortality.

## Data Availability

Statistical data are available from all authors upon request but not publicly available.

## Abbreviations

ANOVA: One-way analysis of variance
baPWV: Brachial-ankle pulse wave velocity
BMI: Body mass index
CABG: Coronary artery bypass grafting
CI: Confidence interval
CKD: Chronic kidney disease
COPD: Chronic obstructive pulmonary disease
ECG: Electrocardiogram
E/e′: Early diastolic transmitral flow velocity to mitral annular tissue velocity
EF: Ejection fraction
eGFR: Estimated glomerular filtration rate
EVAR: Endovascular abdominal aortic repair
IPTW: Inverse probability of treatment weighting
LITA: Left internal thoracic artery
LV: Left ventricular
MI: Myocardial infarction
OPCAB: Off-pump coronary artery bypass grafting
OR: Odds ratio
PMI: Perioperative myocardial infarction
POAF: Postoperative atrial fibrillation
POAKI: Postoperative acute kidney injury
PODs: Postoperative days
POSD: Postoperative stroke/delirium
SD: Standard deviation
SV: Saphenous vein

## References

[1] Cavalcante JL, Lima JA, Redheuil A, Al-Mallah MH (2011). Aortic stiffness: current understanding and future directions. J Am Coll Cardiol.

[2] Mottram PM, Haluska BA, Leano R (2005). Relation of arterial stiffness to diastolic dysfunction in hypertensive heart disease. Heart.

[3] Chow B, Rabkin SW (2015). The relationship between arterial stiffness and heart failure with preserved ejection fraction: a systemic meta-analysis. Heart Fail Rev.

[4] Park JH, Marwick TH (2011). Use and limitations of E/e′ to assess left ventricular filling pressure by echocardiography. J Cardiovasc Ultrasound.

[5] Vlachopoulos C, Aznaouridis K, Stefanadis C (2010). Prediction of cardiovascular events and all-cause mortality with arterial stiffness: a systematic review and meta-analysis. J Am Coll Cardiol.

[6] Metkus TS, Suarez-Pierre A, Crawford TC (2018). Diastolic dysfunction is common and predicts outcome after cardiac surgery. J Cardiothorac Surg.

[7] Choi JS, Oh SJ, Sung YW, Moon HJ, Lee JS (2020). Pulse wave velocity is a new predictor of acute kidney injury development after off-pump coronary artery bypass grafting. PLoS ONE.

[8] Kim HL, Lim WH, Seo JB (2017). Association between arterial stiffness and left ventricular diastolic function in relation to gender and age. Medicine.

[9] Kaw R, Hernandez AV, Pasupuleti V, Cardiovascular Meta-analyses Research Group (2016). Effect of diastolic dysfunction on postoperative outcomes after cardiovascular surgery: a systematic review and meta-analysis. J Thorac Cardiovasc Surg.

[10] Nagueh SF, Smiseth OA, Appleton CP (2016). Recommendations for the evaluation of left ventricular diastolic function by echocardiography: an update from the American Society of Echocardiography and the European Association of Cardiovascular Imaging. J Am Soc Echocardiogr.

[11] Hur M, Nam K, Jo WY, Kim G, Kim WH, Bahk JH (2018). Association between elevated echocardiographic index of left ventricular filling pressure and acute kidney injury after off-pump coronary artery surgery. Circ J.

[12] Kellum JA, Lameire N, Aspelin P (2012). Kidney disease: improving global outcomes (KDIGO) clinical practice guideline for acute kidney injury. Kidney Inter.

[13] Tanaka Y, Shah NS, Passman R, Greenland P, Lloyd-Jones DM, Khan SS (2021). Trends in cardiovascular mortality related to atrial fibrillation in the United States, 2011 to 2018. J Am Heart Assoc.

[14] Prosser J, MacGregor L, Lees KR, Diener HC, Hacke W, Davis S, VISTA Investigators (2007). Predictors of early cardiac morbidity and mortality after ischemic stroke. Stroke.

[15] Greenwood SA, Mangahis E, Castle EM (2019). Arterial stiffness is a predictor for acute kidney injury following coronary artery bypass graft surgery. J Cardiothorac Surg.

[16] Lazzeri C, Valente S, Tarquini R, Gensini GF (2011). Cardiorenal syndrome caused by heart failure with preserved ejection fraction. Int J Nephrol.

[17] Williams B, Lacy PS (2010). Central haemodynamics and clinical outcomes: going beyond brachial blood pressure?. Eur Heart J.

[18] Leite-Moreira AF, Correia-Pinto J, Gillebert TC (1999). Afterload induced changes in myocardial relaxation: a mechanism for diastolic dysfunction. Cardiovasc Res.

[19] Lantelme P, Laurent S, Besnard C (2008). Arterial stiffness is associated with left atrial size in hypertensive patients. Arch Cardiovasc Dis.

[20] Rosenberg MA, Manning WJ (2012). Diastolic dysfunction and risk of atrial fibrillation: a mechanistic appraisal. Circulation.

[21] Melduni RM, Cullen MW (2012). Role of left ventricular diastolic dysfunction in predicting atrial fibrillation recurrence after successful electrical cardioversion. J Atr Fibrillation.

[22] Roach GW, Kanchuger M, Mangano CM, Multicenter Study of Perioperative Ischemia Research Group, Ischemia Research and Education Foundation Investigators (1996). Adverse cerebral outcomes after coronary bypass surgery. N Engl J Med.

[23] Smulter N, Lingehall HC, Gustafson Y, Olofsson B, Engström KG (2013). Delirium after cardiac surgery: incidence and risk factors. Interact Cardiovasc Thorac Surg.

[24] Fontes ML, Aronson S, Mathew JP, Multicenter Study of Perioperative Ischemia (McSPI) Research Group, Ischemia Research and Education Foundation (IREF) Investigators (2008). Pulse pressure and risk of adverse outcome in coronary bypass surgery. Anesth Analg.

[25] Kidher E, Harling L, Sugden C (2014). Aortic stiffness is an indicator of cognitive dysfunction before and after aortic valve replacement for aortic stenosis. Interact Cardiovasc Thorac Surg.

[26] Laugesen E, Olesen KKW, Peters CD (2022). Estimated pulse wave velocity is associated with all-cause mortality during 8.5 years follow-up in patients undergoing elective coronary angiography. J Am Heart Assoc.

[27] Kim JM, Kim SS, Kim IJ (2020). Arterial stiffness is an independent predictor for risk of mortality in patients with type 2 diabetes mellitus: the REBOUND study. Cardiovasc Diabetol.

[28] Okura H, Takada Y, Kubo T (2006). Tissue doppler-derived index of left ventricular filling pressure, E/E', predicts survival of patients with non-valvular atrial fibrillation. Heart.

[29] Ben-Shlomo Y, Spears M, Boustred C (2014). Aortic pulse wave velocity improves cardiovascular event prediction: an individual participant meta-analysis of prospective observational data from 17,635 subjects. J Am Coll Cardiol.

