# Supplementary material for: Combined impact of elevated arterial stiffness and left ventricular filling pressure on outcomes after off-pump coronary artery bypass grafting
Source: J Cardiothorac Surg. 2022 Oct 2;17:248. doi: 10.1186/s13019-022-01994-5 (PMC9528179; doi:10.1186/s13019-022-01994-5)
Supplement: Supplementary file 1 — Additional file 1: Table S1. Postoperative complications. Table S2. Comparison of major postoperative complications across the groups. Table S3. Inter-group comparison with Cox regression analysis for all-cause mortality. Table S4. Cox regression analysis for all-cause mortality. [file 13019_2022_1994_MOESM1_ESM.docx]

**Table S1** Postoperative complications

|  | Before IPTW adjustment | | | | | | After IPTW adjustment | | | | |
| --- | --- | --- | --- | --- | --- | --- | --- | --- | --- | --- | --- |
|  | Total | Reference *^a^* | High-PWV -only | High-E/e' -only | High-PWV -and-E/e' | P | Reference | High-PWV -only | High-E/e' -only | High-PWV -and-E/e' | P |
| N | 164 | 89 | 28 | 29 | 18 |  | 89 | 28 | 29 | 18 |  |
| AKI, n(%) or % | 26 (15.9) | 5 (5.6) | 7 (25.0) | 3 (10.3) | 11 (61.1) | **<0.001** *^b,d,g^* | 5.4 | 19.8 | 7.1 | 52.9 | **<0.001***^d,g^* |
| Stroke/delirium, n (%) or % | 19 (11.6) | 8 (9.0) | 4 (14.3) | 2 (6.9) | 5 (27.8) | 0.133 | 12.9 | 15.9 | 5.8 | 24.0 | 0.319 |
| Atrial fibrillation, n (%) or % | 45 (27.4) | 21 (23.6) | 2 (7.1) | 11 (37.9) | 11 (61.1) | **<0.001** *^d,e,f^* | 23.1 | 7.8 | 27.9 | 68.6 | **<0.001***^d,f,g^* |
| Graft patency,  PA/DA (%) or % | 460/476  (96.6) | 253/260  (97.3) | 78/82  (95.1) | 76/80  (95.0) | 53/54  (98.1) | 0.715 | 97.7 | 94.7 | 96.6 | 98.1 | 0.745 |
| Perioperative MI, n (%) or % | 16 (9.8) | 10 (11.2) | 4 (14.3) | 2 (6.9) | 0 (0.0) | 0.393 | 10.4 | 16.8 | 3.3 | 0.0 | 0.158 |
| IABP support, n (%) or % | 5 (3.0) | 1 (1.1) | 0 (0) | 4 (13.8) | 0 (0.0) | **0.025** | 0.8 | 0.0 | 15.7 | 0.0 | **0.001***^c^* |
| ECMO support, n | 0 | 0 | 0 | 0 | 0 | - | 0 | 0 | 0 | 0 | - |
| Pneumonia, n (%) or % | 13 (7.9) | 5 (5.6) | 2 (7.1) | 4 (13.8) | 2 (11.1) | 0.416 | 4.4 | 4.3 | 13.3 | 9.5 | 0.343 |
| Peak troponin-I, ng/mL | 2.3  (1.0-5.2) | 2.5  (1.1-5.2) | 2.2  (1.3-5.2) | 1.3  (0.6-3.6) | 2.8  (1.3-5.5) | 0.294 | 2.5  (1.0-5.2) | 2.2  (1.3-5.4) | 1.3 (0.6-2.6) | 3.4 (1.1-5.7) | 0.373 |
| Ventilator support, hrs | 18.0  (14.4-22.1) | 16.7  (13.0-20.9) | 18.5  (15.0-22.0) | 19.0  (15.5-24.1) | 23.1  (19.3-26.1) | **0.004***^d^* | 17.7  (14.0-21.0) | 18.5 (15.0-23.7) | 18.9  (15.0-23.7) | 22.0  (19.0-25.6) | 0.533 |
| ICU stay, hrs | 46.0  (28.8-65.1) | 46.0  (26.0-68.0) | 44.0  (29.0-59.5) | 46.0  (26.0-71.0) | 49.5 (38.0-85.5) | 0.545 | 46.0  (28.0-69.5) | 46.0  (26.0-91.0) | 45.0  (26.0-91.0) | 46.0 (37.0-67.0) | 0.807 |
| Hospital stay, days | 9.0  (8.0-13.0) | 8.0  (8.0-12.0) | 9.0 (8.0-12.2) | 10.0  (8.0-13.0) | 10.5  (9.0-19.0) | **0.029***^d^* | 9.0  (8.0-12.0) | 9.0  (8.0-12.0) | 9.0 (8.0-12.0) | 9.0 (9.0-26.0) | 0.079 |
| 30-day mortality, n | 0 | 0 | 0 | 0 | 0 | - | 0 | 0 | 0 | 0 | - |

AKI: acute kidney injury; DA: total number of distal anastomoses; E: early diastolic mitral inflow velocity; e': early diastolic mitral annular tissue velocity; ECMO: extracorporeal membrane oxygenator; IABP: intraaortic balloon pump; ICU: intensive care unit; IPTW: inverse probability of treatment weighting; MI: myocardial infarction; PA: total number of patent anastomoses; PWV: pulse wave velocity.

*^a^* Reference: neither high PWV nor high E/e'.

Significant post-hoc test results: *^b^* High-PWV-only vs. Reference; *^c^* High-E/e'-only vs. Reference; *^d^* High-PWV-and-E/e' vs. Reference; *^e^* High-E/e'-only vs. High-PWV-only; *^f^* High-PWV-and-E/e' vs. High-PWV-only; *^g^* High-PWV-and-E/e' vs. High-E/e'-only.

**Table S2** Comparison of major postoperative complications across the groups

|  |  | Before IPTW adjustment | | After IPTW adjustment | |
| --- | --- | --- | --- | --- | --- |
|  | n (%) | OR (95% CI) | P | OR (95% CI) | P |
| **POAKI** | 26 (15.9) |  | **<0.001** |  | **0.002** |
| Reference *^a^* | 5 (5.6) | 1 |  | 1 |  |
| High-PWV-only | 7 (25.0) | 4.782 (1.335, 17.131) | **0.016** | 6.193 (0.987, 38.860) | 0.052 |
| High-E/e'-only | 3 (10.3) | 1.243 (0.251, 6.148) | 0.789 | 0.963 (0.1121, 8.285) | 0.973 |
| High-PWV-and-E/e' | 11 (61.1) | 22.573 (5.918, 86.105) | **<0.001** | **89.458 (8.493, 942.275)** | **<0.001** |
|  |  | Hosmer-Lemeshow test | 0.850 | Hosmer-Lemeshow test | 0.7663 |
| **POAF** | 45 (27.4) |  | **0.001** |  | **0.004** |
| Reference | 21 (23.6) | 1 |  | 1 |  |
| High-PWV-only | 2 (7.1) | 0.219 (0.046, 1.033) | 0.055 | 0.321 (0.053, 1.937) | 0.216 |
| High-E/e'-only | 11 (37.9) | 2.259 (0.896, 5.693) | 0.084 | 2.119 (0.645, 6.958) | 0.216 |
| High-PWV-and-E/e' | 11 (61.1) | 5.668 (1.896, 16.947) | **0.002** | **12.512 (2.453, 63.830)** | **0.002** |
|  |  | Hosmer-Lemeshow test | 0.779 | Hosmer-Lemeshow test | 0.091 |
| **POSD** | 19 (11.6) |  | 0.102 |  | 0.736 |
| Reference | 8 (9.0) | 1 |  | 1 |  |
| High-PWV-only | 4 (14.3) | 2.066 (0.497, 7.851) | 0.296 | 1.315 (0.278, 5.491) | 0.718 |
| High-E/e'-only | 2 (6.9) | 0.550 (0.087, 2.395) | 0.469 | 0.566 (0.083, 2.623) | 0.483 |
| High-PWV-and-E/e' | 5 (27.8) | 4.115 (1.060, 15.680) | 0.039 | 1.744 (0.296, 10.510) | 0.523 |
|  |  | Hosmer-Lemeshow test | 0.964 | Hosmer-Lemeshow test | 0.480 |

CI: confidence interval; E: early diastolic mitral inflow velocity; e': early diastolic mitral annular tissue velocity; IPTW: inverse probability of treatment weighting; OR: odds ratio; POAF: postoperative atrial fibrillation; POAKI: postoperative acute kidney injury; POSD: postoperative stroke and/or delirium; PWV: pulse wave velocity.

*^a^* Reference: neither high PWV nor high E/e'.

All multivariable logistic regression models were adjusted for diabetes, hypertension, stroke history (this was excluded only for the regression model for POSD), eGFR, LVEF, EuroSCORE II, and CHA2DS2-VASc score. Independent variables were entered into this model by backward elimination (Likelihood Ratio). The Hosmer and Lemeshow Goodness-of-fit test was conducted to assess the lack of fit of the models.

**Table S3** Inter-group comparison with Cox regression analysis for all-cause mortality

| Group comparison | Before IPTW adjustment | | | | After IPTW adjustment | | | |
| --- | --- | --- | --- | --- | --- | --- | --- | --- |
|  | Univariable analysis | | Multivariable analysis | | Univariable analysis | | Multivariable analysis | |
|  | HR (95% CI) | p value*^b^* | HR (95% CI) | p value*^b^* | HR (95% CI) | p value* | HR (95% CI) | p value*^b^* |
| Group |  | 0.001 |  | **0.018** |  | **<0.001** |  | **0.004** |
| High-PWV-only vs. Referencea | 1.299 (0.252, 6.698) | 1.000 | 1.337 (0.256, 6.994) | 1.000 | 0.866 (0.163, 4.614) | 1.000 | 0.882 (0.163, 4.788) | 1.000 |
| High-E/e'-only vs. Reference | 3.748 (1.144, 12.286) | 0.175 | 2.457 (0.719, 8.398) | 0.910 | 1.596 (0.473, 5.387) | 1.000 | 0.984 (0.275, 3.528) | 1.000 |
| High-PWV-and-E/e' vs. Reference | 9.190 (3.004, 28.118) | 0.001 | 6.265 (1.931, 20.327) | **0.013** | 8.466 (3.262, 21.971) | 0.005 | 6.090 (2.001, 18.532) | **0.009** |
| High-E/e'-only vs. High-PWV-only | 2.885 (0.582, 14.298) | 1.000 | 1.838 (0.347, 9.743) | 1.000 | 1.843 (0.306, 11.099) | 1.000 | 1.116 (0.174, 7.168) | 1.000 |
| High -PWV-and-E/e' vs. High-PWV-only | 7.073 (1.501, 33.324) | 0.080 | 4.687 (0.951, 23.101) | 0.346 | 9.777 (1.919, 49.806) | 0.037 | 6.903 (1.247, 38.215) | 0.161 |
| High-PWV-and-E/e' vs. High-E/e'-only | 2.452 (0.850, 7.075) | 0.583 | 2.550 (0.841, 7.730) | 0.588 | 5.304 (1.672, 16.828) | 0.028 | 6.186 (1.693, 22.598) | **0.035** |

*^a^* Reference: neither high PWV nor high E/e'; *^b^* Bonferroni adjusted p-value

**Table S4** Cox regression analysis for all-cause mortality

| Risk factors | Before IPTW adjustment | | | | After IPTW adjustment | | | |  |  |
| --- | --- | --- | --- | --- | --- | --- | --- | --- | --- | --- |
|  | Univariable analysis | | Multivariable analysis | | Univariable analysis | | Multivariable analysis | | | |
|  | HR (95% CI) | P | HR (95% CI) | P | HR (95% CI) | P | HR (95% CI) | P | |  |
| Group |  | 0.001 |  | **0.018** |  | **<0.001** |  | **0.004** | |  |
| Reference *^a^* | 1 |  | 1 |  | 1 |  |  |  | |  |
| High-PWV-only | 1.299 (0.252, 6.698) | 0.754 | 1.337 (0.256, 6.994) | 0.731 | 0.866 (0.163, 4.614) | 0.866 | 0.882 (0.163, 4.788) | 0.885 | |  |
| High-E/e'-only | 3.748 (1.144, 12.286) | 0.029 | 2.457 (0.719, 8.398) | 0.152 | 1.596 (0.473, 5.387) | 0.451 | 0.984 (0.275, 3.528) | 0.981 | |  |
| High-PWV-and-E/e' | 9.190 (3.004, 28.118) | <0.001 | 6.265 (1.931, 20.327) | **0.002** | 8.466 (3.262, 21.971) | <0.001 | 6.090 (2.001, 18.532) | **0.002** | |  |
| Sex, Female | 0.894 (0.360, 2.216) | 0.808 |  |  | 1.081 (0.447, 2.616) | 0.863 |  |  | |  |
| Age | 1.047 (0.997, 1.100) | 0.068 |  |  | 1.044(0.994 1.097) | 0.085 |  |  | |  |
| Age75 | 2.156 (0.835, 5563) | 0.112 |  |  | 2.421 (0.972, 6.027) | 0.058 |  |  | |  |
| Body mass index | 0.853 (0.749, 0.971) | 0.016 |  |  | 0.886 (0.783, 1.002) | 0.054 |  |  | |  |
| Smoking | 1.069 (0.454, 2.519) | 0.879 |  |  | 1.303 (0.570, 2.980) | 0.531 |  |  | |  |
| Obesity | 0.284 (0.096, 0.845) | 0.024 |  |  | 0.389 (0.147, 1.028) | 0.057 |  |  | |  |
| NYHA functional class | 0.999 (0.552, 1.812) | 0.999 |  |  | 0.829 (0.449, 1.531) | 0.549 |  |  | |  |
| Hypertension | 8.680 (1.165, 64.685) | 0.035 | 5.574 (0.710, 43.793) | 0.102 | 3.409 (0.933, 12.454) | 0.064 |  |  | |  |
| Diabetes | 1.852 (0.747, 4.590) | 0.183 |  |  | 1.520 (0.659, 3.507) | 0.326 |  |  | |  |
| Insulin | 1.944 (0.712, 5.309) | 0.194 |  |  | 1.792 (0.615, 5.218) | 0.285 |  |  | |  |
| Dyslipidemia | 1.982 (0.842, 4.669) | 0.117 |  |  | 1.854 (0.813, 4.228) | 0.142 |  |  | |  |
| Cerebrovascular accident | 2.917 (1.177, 7.230) | 0.021 | 2.469 (0.923, 6.606) | 0.072 | 3.172 (1.368, 7.356) | **0.007** | 2.796 (1.081, 7.233) | **0.034** | |  |
| Peripheral arteriopathy | 1.983 (0.835, 4.706) | 0.121 |  |  | 2.814 (1.248, 6.347) | **0.013** | 1.867 (0.731, 4.772) | 0.192 | |  |
| CKD grade ≥4 | 5.237 (2.165, 12.672) | <0.001 | 3.545 (1.392, 9.025) | **0.008** | 4.073 (1.640, 10.115) | **0.003** | 2.611 (0.872, 7.823) | 0.086 | |  |
| COPD | 4.693 (1.574, 13.995) | 0.006 | 6.968 (2.184, 22.232) | **0.001** | 3.275 (1.003, 10.697) | **0.049** | 3.830 (1.061, 13.833) | **0.040** | |  |
| Acute myocardial infarction | 1.300 (0.437, 3.867) | 0.637 |  |  | 1.526 (0.561, 4.149) | 0.408 |  |  | |  |
| Atrial fibrillation | 1.848 (0.015, 13.402) | 0.696 |  |  | 2.930 (0.167, 51.358) | 0.462 |  |  | |  |
| PCI | 1.242 (0.365, 4.223) | 0.729 |  |  | 1.028 (0.289, 3.657) | 0.966 |  |  | |  |
| EuroSCORE II | 1.111 (1.010, 1.223) | 0.031 |  |  | 1.088 (0.965, 1.228) | 0.170 |  |  | |  |
| EuroSCORE II ≥4 | 2.263 (0.878, 5.834) | 0.091 |  |  | 1.432 (0.487, 1.211) | 0.514 |  |  | |  |
| EuroSCORE II > 7 | 3.407 (0.793, 14.633) | 0.099 |  |  | 2.154 (0.362, 12.809) | 0.399 |  |  | |  |
| CHA2DS2-VASc SCORE | 1.100 (1.032, 1.173) | 0.004 |  |  | 1.081 (1.016, 1.151) | **0.013** | 1.023 (0.941, 1.111) | 0.597 | |  |
| Ejection fraction | 0.996 (0.965, 1.028) | 0.825 |  |  | 1.011 (0.979, 1.044) | 0.514 |  |  | |  |
| Pulmonary hypertension | 1.075 (0.979, 1.181) | 0.127 |  |  | 1.089 (0.989, 1.199) | 0.083 |  |  | |  |
| Preoperative IABP | 0.486 (0.004, 3.507) | 0.569 |  |  | 0.405 (0.023, 7.090) | 0.536 |  |  | |  |
| Three vessel disease | 0.571 (0.221, 1.473) | 0.246 |  |  | 0.537 (0.232, 1.245) | 0.147 |  |  | |  |
| Left main disease | 0.921 (0.372, 2.283) | 0.860 |  |  | 0.841 (0.351, 2.013) | 0.697 |  |  | |  |
| Urgency | 1.254 (0.168, 9.349) | 0.825 |  |  | 0.989 (0.096, 10.232) | 0.992 |  |  | |  |

CKD: chronic kidney disease; E: early diastolic mitral inflow velocity; e': early diastolic mitral annular tissue velocity; HR: hazard ratio; IABP: intraaortic balloon pump; IPTW: inverse probability of treatment weighting; OR: odds ratio; PCI: percutaneous coronary intervention; PWV: pulse wave velocity.

*^a^* Reference: neither high PWV nor high E/e'.

The covariates included in the multivariable models were selected based on statistical significance in univariable analysis.
